# Cost-Effectiveness of Monovalent Rotavirus Vaccination of Infants in Malawi: A Postintroduction Analysis Using Individual Patient–Level Costing Data

**DOI:** 10.1093/cid/civ1025

**Published:** 2016-04-07

**Authors:** Naor Bar-Zeev, Jacqueline E. Tate, Clint Pecenka, Jean Chikafa, Hazzie Mvula, Richard Wachepa, Charles Mwansambo, Themba Mhango, Geoffrey Chirwa, Amelia C. Crampin, Umesh D. Parashar, Anthony Costello, Robert S. Heyderman, Neil French, Deborah Atherly, Nigel A. Cunliffe

**Affiliations:** 1 Malawi-Liverpool-Wellcome Trust Clinical Research Programme, College of Medicine, University of Malawi, Blantyre; 2 Institute of Infection and Global Health, University of Liverpool, United Kingdom; 3 Epidemiology Branch, Division of Viral Diseases, National Center for Immunization and Respiratory Diseases, Centers for Disease Control and Prevention, Atlanta, Georgia; 4 Program for Appropriate Technologies in Health (PATH), Seattle, Washington; 5 Karonga Prevention Study, Chilumba, Karonga; 6 Ministry of Health, Lilongwe, Malawi; 7 London School of Hygiene and Tropical Medicine; 8 Institute of Global Health; 9 Division of Infection and Immunity, University College London; 10 Liverpool School of Tropical Medicine, United Kingdom

**Keywords:** rotavirus vaccine, cost-effectiveness, developing countries

## Abstract

***Background.*** Rotavirus vaccination reduces childhood hospitalization in Africa, but cost-effectiveness has not been determined using real-world effectiveness and costing data. We sought to determine monovalent rotavirus vaccine cost-effectiveness in Malawi, one of Africa's poorest countries and the first Gavi-eligible country to report disease reduction following introduction in 2012.

***Methods.*** This was a prospective cohort study of children with acute gastroenteritis at a rural primary health center, a rural first referral–level hospital and an urban regional referral hospital in Malawi. For each participant we itemized household costs of illness and direct medical expenditures incurred. We also collected Ministry of Health vaccine implementation costs. Using a standard tool (TRIVAC), we derived cost-effectiveness.

***Results.*** Between 1 January 2013 and 21 November 2014, we recruited 530 children aged <5 years with gastroenteritis. Costs did not differ by rotavirus test result, but were significantly higher for admitted children and those with increased severity on Vesikari scale. Adding rotavirus vaccine to the national schedule costs Malawi $0.42 per dose in system costs. Vaccine copayment is an additional $0.20. Over 20 years, the vaccine program will avert 1 026 000 cases of rotavirus gastroenteritis, 78 000 inpatient admissions, 4300 deaths, and 136 000 disability-adjusted-life-years (DALYs). For this year's birth cohort, it will avert 54 000 cases of rotavirus and 281 deaths in children aged <5 years. The program will cost $10.5 million and save $8.0 million in averted healthcare costs. Societal cost per DALY averted was $10, and the cost per rotavirus case averted was $1.

***Conclusions.*** Gastroenteritis causes substantial economic burden to Malawi. The rotavirus vaccine program is highly cost-effective. Together with the demonstrated impact of rotavirus vaccine in reducing population hospitalization burden, its cost-effectiveness makes a strong argument for widespread utilization in other low-income, high-burden settings.

Rotavirus gastroenteritis is a leading cause of illness and death in African children, accounting for >197 000 deaths annually, just over half of the global rotavirus mortality burden [1, 2]. Since 2012, with Gavi, the Vaccine Alliance (hereafter “Gavi”) support, 25 African countries have introduced rotavirus vaccine into their childhood immunization programs. New vaccines are costly to the health system, and the expenditure should be justified on epidemiological and fiscal grounds as investment in vaccine programs necessarily denies funds from competing health priorities. Additionally, as Gavi-supported countries are required to make copayments for vaccines, knowing these vaccines are cost-effective is important for budgetary planning and negotiating procurement costs. Cost-effectiveness is evaluated in terms of gross domestic product (GDP) and thus is context dependent, both with respect to program and treatment costs but also with respect to local vaccine effectiveness. According to World Health Organization (WHO) criteria, cost per disability-adjusted life-year (DALY) [3] ratios <3 times the per-capita GDP are considered cost-effective, whereas those less than per-capita GDP are considered highly cost-effective [4]. (DALY measures the population loss of years of life lived in perfect health.) Estimates from middle- or high-income settings are therefore not applicable to low-income countries.

Malawi, a low-income country in southern Africa with under-5 mortality of 71 per 1000 live births and GDP per capita of $253 [5], was one of the first countries on the continent to introduce monovalent rotavirus vaccine in 2012. The government of Malawi is the main healthcare provider in the country and healthcare is free at government facilities, although families often incur considerable ancillary costs and there is no national health insurance scheme. We recently described monovalent rotavirus vaccine effectiveness against severe acute rotavirus gastroenteritis of 64% (95% confidence interval, 24%–83%) in routine use in Malawi [6]. Previous cost-effectiveness studies from Malawi and elsewhere in Africa conducted prior to vaccine introduction were based on modeled (top-down) estimates of cost rather than empirically observed (ground-up) actual expenditures [7–9]. We now report results from a comprehensive individual patient costing cohort study using actual ongoing costs incurred by the health system and by households to determine, from a government and from a societal perspective, rotavirus vaccine cost-effectiveness following introduction.

## METHODS

### Study Sites and Design

Between 1 January 2013 and 21 November 2014, we conducted a prospective cohort study of children <5 years of age resident within the study site catchment areas presenting with acute gastroenteritis in northern and southern Malawi. The northern rural site was located in Chilumba Rural Hospital campus, Karonga, on which site are colocated an “under-5” primary health center providing childhood outpatient services and, adjacently housed, a first-referral-level inpatient facility. Both service the population within a demographic surveillance site in Chilumba that has been described previously [10, 11]. The southern urban site was at Queen Elizabeth Central Hospital, Blantyre, which is the tertiary referral center for Malawi's south and serves as a district hospital for the city of Blantyre (population 1.3 million). All sites are government facilities providing free healthcare. At these sites, with parental written consent, we recruited both outpatient and admitted children. We excluded children admitted to another hospital >24 hours who then subsequently transferred to study facilities, children re-presenting for the same illness within 14 days, and children with known oncological or congenital immunodeficiency other than human immunodeficiency virus infection. Demographic and clinical data were collected at enrollment (Table 1) and disease severity on admission was measured by Vesikari score, with severe disease defined as a score >10. Stool specimens were tested for rotavirus using enzyme immunoassay (Rotaclone, Meridian Bioscience, Cincinnati, Ohio). We followed up children surviving to facility discharge with a home visit after 6 weeks to obtain all illness-related costs incurred after discharge. Initial training of interviewers was exhaustive and included role-play scenarios as well as peer review to ensure consistency in data recording and interpretation of parental answers regarding income and expenditures.

**Table 1.** Cohort Demographics

Characteristic	Urban			Rural		
	Inpatient (n = 282)	Outpatient (n = 118)	Total (n = 400)	Inpatient (n = 22)	Outpatient (n = 108)	Total (N = 130)
Age						
0–11 mo	151 (54)	42 (36)	193 (48)	11 (50)	53 (49)	64 (49)
12–23 mo	105 (37)	51 (43)	156 (39)	5 (23)	26 (24)	31 (24)
24–59 mo	24 (9)	17 (14)	41 (10)	4 (18)	26 (24)	30 (23)
Mean (SD), mo	13.6 (8.0)	17.9 (13.0)	14.8 (9.8)	16.2 (14.2)	15.9 (11.4)	15.9 (11.8)
Male sex	168 (60.0)	63 (53)	231 (58)	9 (41)	62 (57)	71 (55)
Persons in household, median (IQR)	5 (4–6)	4 (3–5)	5 (3.5–6)	6 (4–7)	6 (5–7)	6 (4–7)
Transport to facility						
Walk	15 (5)	17 (14)	32 (8)	10 (46)	59 (55)	69 (53)
Bicycle	1 (0.4)	0	1 (0.3)	10 (46)	36 (33)	46 (35)
Car	19 (7)	1 (1)	20 (5)	0	3 (3)	3 (2)
Minibus	214 (76)	84 (71)	298 (75)	0	0	0
Other	18 (6)	36 (31)	54 (14)	2 (9)	10 (9)	12 (9)
Water source						
Piped to house	38 (14)	23 (20)	61 (15)	2 (9)	14 (13)	16 (12)
Communal piped tap	187 (66)	86 (73)	273 (68)	3 (14)	16 (15)	19 (15)
Borehole	35 (12)	5 (4)	40 (10)	7 (32)	31 (29)	38 (29)
Protected well	3 (1)	0	3 (1)	0	5 (5)	5 (4)
Open well	11 (4)	1 (1)	12 (3)	4 (18)	2 (2)	6 (5)
Open lake/stream	0	0	0	0	1 (1)	1 (1)
Toilet facilities						
Flush toilet	12 (4)	6 (5)	18 (5)	0	0	0
Improved latrine	0	1 (1)	1 (0.3)	0	0	0
Pit latrine	261 (93)	106 (90)	367 (92)	16 (73)	58 (54)	74 (57)
Open	2 (1)	1 (1)	3 (1)	6 (27)	50 (46)	56 (43)
Handwashing facilities available	85 (30)	71 (60)	156 (39)	4 (18)	23 (21)	27 (21)
Caregiver						
Mother	274 (97)	118 (100)	392 (98)	20 (91)	102 (94)	122 (94)
Education of caregiver						
Tertiary	17 (6)	3 (3)	20 (5)	0	1 (1)	1 (1)
Secondary	113 (40)	64 (54)	177 (44)	5 (23)	28 (26)	33 (25)
Primary	122 (43)	46 (39)	168 (42)	15 (68)	76 (70)	91 (70)
None	6 (2)	1 (1)	7 (2)	0	0	0
Unknown	15 (5)	3 (3)	18 (5)	0	0	0
Profession of caregiver						
Housework/child care	157 (56)	68 (58)	225 (56)	12 (55)	41 (38)	53 (41)
Farming	7 (3)	2 (2)	9 (2)	6 (27)	4 (4)	10 (8)
Small business/self-employed	75 (27)	30 (25)	105 (26)	2 (9)	2 (2)	4 (3)

Data are presented as No. (%) unless otherwise specified.

Abbreviations: IQR, interquartile range; SD, standard deviation.

### Data on Cost of Illness

Using a standard case report form, we undertook detailed itemized individual patient–level determination of actual expenditures related to the current illness. From a household perspective, we collected by parental interview self-reported recall of all illness-related expenditures from symptom onset to convalescence at home after discharge, including transport costs to and from health facilities, loss of income and other opportunity costs, costs of formal and informal healthcare seeking (including household expenditure on consultations, diagnostics, and therapeutics in community healthcare facilities, traditional healers, or other informal care), and costs related to accommodation and food for visiting family members during admission of the index child and any direct medical costs. We did not include long-run costs arising from any disability related to the acute illness episode.

From a government healthcare provider perspective, we collected actual costs incurred in our cohort (Table 2). From the medical record we obtained individual-level drugs dispensed and laboratory or radiological investigations performed. Use of clinical consumables (intravenous cannula, stationery, etc) was difficult to account for per child and thus was not included, although our experience in these facilities suggests these costs are likely to be minimal. Drug consumption was costed based on actual procurement purchase costs obtained from the purchasing officer. Laboratory investigations were costed on basis of actual charge incurred by the respective facilities for each specific investigation performed; this charge was inclusive of laboratory consumables, staff time, etc. Costs for each cadre of staff were calculated by multiplying actual salaries (including oncosts such as medical benefits scheme contributions, study leave, other allowances, etc) of staff in observed ward attendance divided by ward occupancy and multiplied by individual patient length of stay. We did not include staff time not spent on direct patient care (eg, in-service training). Hotel costs (ie, kitchen, laundry, sanitation, security, amenities, and transportation) were based on actual hospital expenditures per bed multiplied by individual length of stay. Hospital administration costs, capital costs, or physical asset depreciation were not included. Household costs included all illness-related expenditures that were incurred at each health facility attended, such as direct medical costs (consultation fees, drug cost, diagnostic test cost), transport cost, and any other costs relating to illness as reported by the respondent. We collected costs of accommodation, food, and any other items (soap, cup, etc) for all participants, although only inpatients reported such costs. Opportunity costs (eg, lost income) were included for all participants regardless of which facility they attended. The combination of healthcare costs and household costs over all projected birth cohorts constitute the total societal cost. All costs were collected in 2014 Malawi kwacha, which were converted to US dollars based on the Reserve Bank of Malawi midmarket exchange rate as of 15 July 2014 (Table 2).

**Table 2.** Input Parameters for Estimating Health Service Costs, and Per-Visit Costs by Admission Status and Disease Severity

Sector in Which Cost Incurred	No.	Estimate	95% CI	No.	Estimate	95% CI	Rank-Sum *P* Value
Government cost overall	Outpatient			Inpatient			
Public health center	108	$8.02	$7.47–$8.57	22	$55.04	$43.15–$66.93	<.001
Public tertiary referral hospital	118	$7.15	$6.41–$7.90	282	$47.16	$40.65–$53.67	<.001
Government cost overall	Nonsevere disease			Severe disease			
Public health center	128	$15.50	$11.92–$19.08	2	$46.34^a^	$0–$357.84	.05
Public tertiary referral hospital	197	$26.22	$17.86–$34.57	207	$43.59	$38.54–$48.64	<.001
Government cost for outpatient visit							
Public health center	108	$8.02	$7.47–$8.57	0^b^	…	…	.12^c^
Public tertiary referral hospital	116	$7.02	$6.32–$7.72	2	$14.85	$0–$120.79	.08
Government cost for inpatient admission							
Public rural hospital	20	$55.91	$43.32–$68.49	2	$46.34	$0–$357.84	.65^d^
Public tertiary referral hospital	77	$55.90	$36.07–$75.73	205	$43.87	$38.79–$48.96	.25^d^
Household cost overall	Outpatient			Inpatient			
Public health center^e^	108	$0.49	$0.30–$0.68	22	$9.43	$4.96–$13.89	<.001
Public tertiary referral hospital	118	$5.80	$3.93–$7.68	282	$10.76	$9.38–$12.13	<.001
Household cost overall	Nonsevere disease			Severe disease			
Public health center	128	$1.81	$0.92–$2.71	2	$14.23	$0–$100.57	.015
Public tertiary referral hospital	197	$7.69	$6.17–$9.21	207	$10.94	$9.29–$12.59	<.001
Household cost for outpatient visit							
Public health center^e^	108	$0.49	$0.30–$0.68	0	…	…	.56^c^
Public tertiary referral hospital	116	$5.80	$3.93–$7.68	2	$6.82	$0–$21.26	.23
Household cost for inpatient admission							
Public rural hospital	20	$8.95	$4.16–$13.73	2	$14.23	$0–$100.57	.25
Public tertiary referral hospital	77	$10.16	$7.75–$12.56	205	$10.98	$9.31–$12.65	.38
Other household costs							
Private pharmacy		$0.23	$0.09–$0.36		$0.28	$0.10–$0.46	
Private clinic		$0.04	$0.01–$0.07		$0.13	$0.00–$0.27	

All costs are in 2014 US dollars.

Abbreviation: CI, confidence interval (estimate ± 1.96 × standard error).

^a^ All severe cases in rural setting were admitted to the rural hospital on site (these costs were not entered twice in the cost-effectiveness model).

^b^ Cases with severe disease were admitted after first being seen in outpatient clinic. Subsequent costs of severe disease when admitted were counted under inpatient admission and not outpatient visit. Outpatient visits occurred at public health center and at the outpatient department of the public referral hospital. Inpatient admissions occurred at the public rural hospital and the public referral hospital.

^c^ Linear regression of cost vs Vesikari score for those with score <10.

^d^ Nonsignificant but higher cost point estimate in nonsevere group, possibly explained by admission indicated by other comorbidity rather than gastroenteritis severity itself.

^e^ Total outpatient costs, including costs of healthcare sought before arrival at recruitment facility.

### Data on Vaccine Program Cost

Ongoing rotavirus vaccine program cost projections were provided by the 2012–2016 comprehensive Expanded Programme on Immunization (EPI) multiyear plan [12]. Cost categories included staff training, community sensitization, surveillance, management, transport, maintenance, and capital costs (such as cold storage and transport). We did not include depreciation of capital costs. Personnel costs were excluded because no additional EPI staff were employed with the introduction of rotavirus vaccine, and all work was absorbed by existing staff. We used the mean expenditure over a 5-year period to represent the average annual cost we would expect over the life of the program. These costs were divided by total doses required by the birth cohort, and the cost share was allocated to rotavirus on a per-antigen-dose basis to obtain a system cost per dose as 2014 Malawi kwacha. Our calculations indicate that the addition of rotavirus vaccine to the routine EPI schedule increases system costs by $0.42 per dose. Malawi's copayment is $0.20 [13], with the remainder of the $2.50 dose cost being borne by Gavi. As sensitivity analysis, we modeled Malawi taking on the full cost of $2.50 from both 2023 and 2028, respectively 10 and 15 years after introduction.

### Data on Disease Burden and Vaccine Effectiveness

Disease burden estimates were based on our observed incidence in Malawi and other published burden estimates from Africa [14, 15]; we assumed no changes in quality or availability of clinical care or of population nutrition over time (Table 3). Vaccine coverage and timeliness were those empirically observed in our surveillance program (Table 3) [6]. We used our recently published estimate of vaccine effectiveness against severe disease in Malawi [6], but input lower effectiveness against mild disease based on published estimates and an unpublished systematic review of effectiveness studies [14] (Dan Hungerford, personal communication). We used published case fatality estimates from regional countries, and these were consistently within the error margin of our own measured fatality rate (Table 3) [2, 11, 16]. Because evidence indicates that monovalent rotavirus vaccine provides heterotypic (cross-genotype) protection [20], we assumed no impact on effectiveness over time from genotype replacement in primary analysis, but allowed for this in sensitivity analysis. We assumed waning immunity beyond the first year of life, but modeled unchanging ongoing protective immunity in sensitivity analysis [18, 19]. Disability weighting for DALY calculations was based on published estimates (Table 3) [17].

**Table 3.** Input Parameters for Estimating Disease Burden, Vaccine Coverage, Timeliness, and Effectiveness

Parameter	Estimate	Source(s)
Annual incidence per 100 000 aged 1–59 mo		
Rotavirus (nonsevere) cases, No.	9201	Assumption, derived from [14, 15]
Rotavirus (severe) cases, No.	799	Assumption, derived from [14, 15]
Rotavirus case fatality rate^a^	4.29%	Assumption, derived from [2, 11, 16]
Disability weight for DALY calculations		
Rotavirus (nonsevere) cases	0.202	[17]
Rotavirus (severe) cases	0.281	[17]
Mean duration of illness, d		
Rotavirus (nonsevere) cases	6	[6]
Rotavirus (severe) cases	6	[6]
Age distribution of disease cases and deaths		
<3 mo	6.6%	[6]
3–5 mo	19.4%	[6]
6–8 mo	31.9%	[6]
9–11 mo	19.8%	[6]
12–23 mo	21.8%	[6]
24–35 mo	0.5%	[6]
36–47 mo	0%	[6]
48–59 mo	0%	[6]
Location of care seeking		
Private pharmacy/clinic	15%	Self-reported by this study cohort
Public/government primary health center	70%	Self-reported by this study cohort
Public/government first-level hospital	10%	Self-reported by this study cohort
Public/government referral-level hospital	5%	Self-reported by this study cohort
Total coverage in first year following introduction		
RV1 dose 1	90.2%	[6]
RV1 dose 2	86.9%	[6]
Coverage of dose 1 achieved by age in first year following introduction^b^		
3 mo	75.8%	[6]
6 mo	89.4%	[6]
9 mo	89.9%	[6]
12 mo	89.9%	[6]
Coverage of dose 2 achieved by age in first year following introduction^b^		
3 mo	32.2%	[6]
6 mo	76.0%	[6]
9 mo	84.5%	[6]
12 mo	86.3%	[6]
VE of 2 doses^c^ vs rotavirus (severe) cases		
	64% (95% CI, 24%–83%)	[6]
VE of 2 doses^c^ vs rotavirus (nonsevere) cases		
	40% (95% CI, 30%–60%)	Assumption, derived from [6]
Other vaccination impact assumptions		
% decrease in dose effectiveness per year	47.5% (95% CI, 35.7%–59.4%)	[18, 19]

Abbreviations: CI, confidence interval; DALY, disability-adjusted life-year; RV1, monovalent rotavirus vaccine; VE, vaccine effectiveness.

^a^ Derived from diarrheal disease mortality estimate for Malawi [6]. In the absence of vaccination, this ratio is assumed to decline in each successive birth cohort in line with the general trend in mortality among children aged <5 years. This is done by assuming that the fraction of deaths in the under-5 population caused by the disease remains fixed over time.

^b^ Coverage projections over the period 2013–2033 were estimated by assuming rotavirus vaccine will achieve the same coverage and timeliness as diphtheria-tetanus-pertussis vaccine, and by assuming a 5% annual decrease in the gap between final coverage in the cohort (coverage by age 24 mo) and a ceiling of 99.5% (RV1 dose 1) and 98.8% (RV1 dose 2).

^c^ Effectiveness of single dose input at half that of 2 doses.

### Statistical Analysis

Sample size was specifically calculated for this costing study. Taking a healthcare provider perspective, using predefined precision about a continuous cost estimate, a sample size of 88 provided a diarrheal illness cost estimate with a margin of error of ≤10%, assuming a coefficient of variation of 0.5 and at least 1000 children with diarrhea presenting to our study site annually [21–23]. Larger samples provide more precise cost estimates. Mean (therefore total) costs and 95% confidence bounds were reported for households and healthcare provider costs [24]. These analyses were done using Stata software, version 13.1 (StataCorp, College Station, Texas). Vaccine cost-effectiveness was calculated using TRIVAC 2.0 (Pan-American Health Organization), extensive details of which have been published previously [14, 25]. In brief, TRIVAC uses input demographic and disease burden data, vaccine cost, and coverage and effectiveness estimates, as well as user input healthcare utilization and costs to determine incremental cost-effectiveness over 20 stacked under-5 cohorts to derive years of life lost and cost per DALY gained for each cohort's life expectancy at birth [14]. We used the projected annual number of live births and age-specific population projections for Malawi from United Nations Population Division projections 2012 to determine the number of live births in 2013 as 651 684 [26]. We chose to model costs and benefits over a 20-year horizon to assist policy makers in assessing the long-term implications of their decisions. Future costs and benefits were discounted to 2014 levels at 3%.

### Sensitivity Analyses

We conducted univariate sensitivity analysis on variables that impacted our TRIVAC model results most dramatically (Table 4). These variables included cessation (rather than long-term continuation) of Gavi support 10 or 15 years after introduction (but assuming unchanged vaccine cost), increased systems costs, absence of waning immunity beyond the first year of life, lower vaccine effectiveness over time from rotavirus genotype changes, lower case fatality rate, and lower costs of rotavirus care. Details of specific scenarios are outlined in Table 4.

**Table 4.** Discounted Cost-Effectiveness of Rotavirus Vaccine (20 Cohorts Vaccinated During the Period 2014–2033)

Scenario	Government Perspective	Societal Perspective
Cost-effectiveness threshold		
1 × GDP per capita (2014)–WHO threshold for “highly cost-effective” [4]	$253	$253
3 × GDP per capita (2014)–WHO threshold for “cost-effective” [4]	$759	$759
Cost-effectiveness compared to no vaccine		
Net cost of vaccine and related program costs	$2 529 646	$1 308 333
Costs of vaccine program	$10 528 367	$10 528 367
Health service costs avoided	$7 998 721	$9 220 034
DALYs averted	136 290	136 290
US$ per DALY averted	$19	$10
Univariate sensitivity analyses (in US$ per DALY averted)		
Gavi withdraws support in 2023	$161	$152
Gavi withdraws support in 2028	$88	$79
Increased systems cost of 25% to $0.53 per dose	$32	$23
No waning immunity in second year of life	$2	Cost-saving^a^
Genotype changes lowering VE 15 percentage points	$160	$150
Lower cost of rotavirus care (lower bound of 95% CI in Table 2)	$24	$18
Case fatality rate 2%.5%	$32	$16

Costs and DALYs are discounted at 3% per year.

Abbreviations: CI, confidence interval; DALY, disability-adjusted life-year; GDP, gross domestic product; VE, vaccine effectiveness; WHO, World Health Organization.

^a^ This scenario is cost saving to a total of $1.29 million.

## RESULTS

We recruited a total of 530 children with gastroenteritis, comprising 118 outpatient and 282 children admitted at Queen Elizabeth Central Hospital (urban setting), and 108 outpatient and 22 children admitted at Chilumba Rural Hospital (rural setting) (Table 1). Of these 530 children, 71 had rotavirus confirmed by enzyme immunoassay. Costs of illness did not differ by rotavirus status, but from both a government healthcare provider perspective and a household perspective, costs of inpatient care were greater, as were costs of severe illness (Table 2).

Given model assumptions (Tables 1 and 2), we project that over 20 years the rotavirus vaccine program will avert approximately 1 026 000 cases of rotavirus gastroenteritis, 78 000 inpatient admissions, and 4300 deaths. Additionally, the rotavirus vaccine program was projected to avert about 136 000 DALYs. For the cohort born in 2015, this would translate to 54 000 cases of rotavirus gastroenteritis averted and 281 fewer deaths before the cohort reaches 5 years of age. Health and economic projections in absence and presence of vaccine are shown in Table 5. The resultant cost-effectiveness is shown in Table 4. The cost of the rotavirus vaccine program was calculated as just over $10.5 million. The total projected direct healthcare costs for gastroenteritis averted by vaccination were $8.0 million, and total society costs averted were $9.2 million. Thus, government net cost is just under $2.5 million and the societal net cost (the cost of the vaccine program minus the societal cost of illness that is averted by the vaccination program) over the same period is $1.3 million. The cost per DALY averted was $19 from a healthcare provider perspective and $10 from a societal perspective, and the cost per rotavirus case averted was $2 and $1, respectively. Thus, from both the healthcare provider and societal perspectives, in Malawi the rotavirus vaccine program is highly cost-effective.

**Table 5.** Health and Economic Benefits (20 Cohorts Vaccinated During the Period 2014–2033)

Cases and Costs	No Vaccine	With Vaccine	Averted
Total rotavirus cases <5 y	5 303 276	4 277 313	1 025 922
Total severe cases <5 y	423 581	298 721	124 860
Total deaths <5 y	14 671	10 358	4313
DALY lost	464 990	328 700	136 290
YLD - DALY due to morbidity	2120	1730	380
YLL - DALY due to mortality	462 880	326 970	135 910
Total government health services costs	$34 857 067	$26 860 346	$7 998 716
Total outpatient visit costs	$22 765 954	$18 331 365	$4 434 584
Total inpatient admission costs	$12 091 113	$8 528 981	$3 564 132
Total societal health services costs	$39 572 280	$30 352 346	$9 220 034
Total outpatient visit costs	$24 637 042	$19 819 510	$4 817 531
Total inpatient admission costs	$14 935 238	$10 532 736	$4 402 503

Health benefits and costs are discounted at 3% per year.

Abbreviations: DALY, disability-adjusted life-year; YLD, years of life lost to disability; YLL, years of life lost.

### Sensitivity Analyses

In all but 2 scenarios tested in sensitivity analyses, rotavirus vaccination remained highly cost-effective [4]. Although under an assumption of no waning in immunity beyond the first year of life, the vaccine was cost-saving from a societal perspective, saving Malawi >$1 million over 20 years (Table 4).

## DISCUSSION

Rotavirus vaccine has been projected to be highly cost-effective in the world's poorest countries [27, 28], and its introduction is now under way in many low-income countries with support from Gavi. Studies in sub-Saharan Africa have recently established this vaccine's effectiveness and impact on population burden of disease [6, 29]. These welcome benefits, however, must be judged relative to other potential uses of limited health resources, and so establishing this vaccine's cost-effectiveness and budget impact is crucial. Available cost-effectiveness estimates for Malawi, Uganda, and Kenya were based on assumed rather than observed vaccine effectiveness and on modeled rather than empirically observed costs of illness [7–9]. Additionally, these studies did not include household-level costs in the estimation of cost-effectiveness. In this comprehensive itemized study of actual costs of medically attended gastroenteritis treatment and vaccination, the cost to government of providing free outpatient and especially inpatient care is substantial, and our empirically observed costs are higher than previous projected model-based estimates [9]. An intervention is generally considered highly cost-effective if it costs less than the per-capita GDP (Table 4) [4]. Malawi has among the lowest per-capita GDP in the world, yet even under such challenging economic conditions we have shown that rotavirus vaccine is highly cost-effective.

This study carefully and comprehensively collected actual costs incurred by government in vaccine implementation. A study that projected implementation costs and healthcare provider treatment costs, but did not include household costs, found the program to be relatively expensive for Malawi at $18.5 million over 5 years [30]. Using similar methods but with actual expenditures, our cost projection over 20 years was $10.5 million for the vaccination program. Although it is possible we underestimated program costs, ours were ministry-budgeted expenditures and not modeled projections. Whereas the cost of the vaccination program is substantial, it is relatively small in relation to the $429 million in total health expenditure in Malawi in 2013 [31]. Health expenditure in Malawi has more than doubled over the last decade, so it is critical that policy makers choose highly cost-effective health interventions to maximize the impact of these investments.

We made every attempt to provide as accurate an estimate of staff costs as was feasible. But we were unable to cost individual minutes spent by staff in direct patient care, nor were we able to include a patient complexity weighting [32]. However, consistent with a previous study from a tertiary hospital in an affluent area of Johannesburg [33], we did not find evidence of cost differential by rotavirus status. We did find higher costs for severe disease and for inpatient care, but notably disease severity and admission status were closely related (Table 2). Regardless of diarrhea etiology, children are provided empiric treatment regimens in accordance with a standard protocol based on WHO guidelines for dehydration [34], and admitted children have similar length of stay.

Costs for families were based on reported recall, and these estimates could not be externally validated, so are subject to recall biases. However, we did conduct home visits and could confirm other socioeconomic covariates by direct observation, and interviewers had extensive training on appropriate questioning and follow-up prompting of responses to obtain and confirm information by parents. We suspect that the poorer the household, the greater the risk of presenting with diarrheal disease. This is an ascertainment bias of sorts, but also reflects a reality that vaccination may be most important for the most impoverished families, and is likely one of the more equitable health interventions [35–37]. We have estimated the household costs of children attending care, but could not estimate costs to those families too poor to afford access to care. We have, for instance, recently shown that in the Karonga study site, distances to road and health facility are associated with delayed vaccine uptake or nonreceipt of vaccines, and we speculate that the same may be true for care seeking during illness. It is therefore plausible that for the most destitute, costs of attending healthcare are prohibitive, and the very poorest children may be unable to attend care. This would bias our findings. We only recruited children presenting for care, so have not included costs to households of gastroenteritis episodes managed at home. The latter could only be obtained through a large population cohort design, but the exclusion of such costs from our analysis makes our estimate of cost-effectiveness more conservative.

We used our observed population coverage rates and included adjustment for effective coverage—that is, the coverage in those at risk of disease relative to coverage in the entire birth cohort (ie, overall coverage). We did not take into account costs of possible secondary household cases linked to our index patients, nor did we take into account indirect benefits of vaccination in averting secondary cases. If such indirect effects will occur in Malawi as they have elsewhere [33], this would substantially increase the vaccine's cost-effectiveness. Should the burden of disease decline because of socioeconomic improvements over time, then our model may overestimate long-term cost-effectiveness. In sensitivity analyses we found that even in many such circumstances the program remains highly cost-effective. Forecasting the impact of future scenarios on illness costs is conjectural at best, so our sensitivity analyses should be interpreted with due caution. The purpose of these analyses was not to predict future costs with precision, but to test whether cost-effectiveness, broadly speaking, is maintained. In this regard the outcome was consistently affirmative. Over time, countries will increasingly be required to bear a higher copayment for vaccine procurement, though cost of vaccine is likely to decline over time. Although Malawi does not appear to face an imminent increase in its copayment, even were this to happen at current prices, cost-effectiveness would be maintained, perhaps even enhanced should future prices decline further once additional products are marketed.

Although this study included primary healthcare, first-referral-level, and tertiary-level facilities in urban and rural settings and included inpatients and outpatients, caution is warranted in extrapolating our findings to other settings, particularly those in which vaccine effectiveness has not been evaluated, or whose healthcare systems differ substantially from Malawi. In addition, the very presence of our longstanding research activities at our recruitment sites may reduce applicability to other government clinics where research and, by implication, an emphasis on good quality clinical care are enhanced. Malawi's population utilization of primary and subsequent referral-level care was uncertain; thus, we assumed proportions based on healthcare utilization reported by our cohort. We ran our models with differing but plausible distributions, and this did not dramatically affect cost-effectiveness estimates. We therefore believe it is likely that in many resource-limited settings our findings have applicability, but clearly local health planners should be mindful of their own settings when considering the relevance of our findings. Model-based expectations of cost-effectiveness have been reported from Southeast Asia, where the vaccine is yet to be introduced [28].

The Malawian economy underwent a period of substantial instability in the wake of the devaluation of the kwacha in 2012. This ushered in inflation and marked increases in costs of goods and services. Inflation had stabilized by the time we embarked on the bulk of our recruitment, but the uncertain economic climate persists. Such turbulence is difficult to adjust for in analysis, but is all too common in low-income countries. Indeed, such instabilities are often associated with increased illness, thereby strengthening further the argument for introducing and maintaining vaccine programs that are effective and cost-effective even in the short run.

In conclusion, gastroenteritis episodes represent a substantial economic burden to government and to families. We found monovalent rotavirus vaccine to be highly cost-effective in Malawi, even under challenging possible future scenarios. Together with the demonstrated impact of monovalent rotavirus vaccine in reducing population hospitalization burden, the additional cost savings afforded by this vaccine make a strong argument for the widespread introduction in other low-income, high-burden settings.
